# Cardiovascular outcomes with SGLT2 inhibitors versus DPP4 inhibitors and GLP-1 receptor agonists in patients with heart failure with reduced and preserved ejection fraction

**DOI:** 10.1186/s12933-023-01784-w

**Published:** 2023-03-10

**Authors:** Jimmy Gonzalez, Benjamin A. Bates, Soko Setoguchi, Tobias Gerhard, Chintan V. Dave

**Affiliations:** 1 Department of Pharmacy Practice and Administration, Ernest Mario School of Pharmacy, Rutgers University, Piscataway, NJ USA; 2 Department of Pharmacy, Jersey Shore University Medical Center, Neptune, NJ USA; 3 Center for Pharmacoepidemiology and Treatment Science, Institute for Health, Health Care Policy and Aging Research, Rutgers University, 112 Paterson St, New Brunswick, NJ 08901 USA; 4 Department of Medicine, Rutgers Robert Wood Johnson Medical School, New Brunswick, NJ USA

**Keywords:** Sodium–glucose cotransporter-2 inhibitors, Glucagon-like peptide-1 receptor agonists, Cardiovascular outcomes, Major adverse cardiovascular events, Heart failure, Stroke, Myocardial infarction, Hospitalization for heart failure

## Abstract

**Background:**

No study has compared the cardiovascular outcomes for sodium–glucose cotransporter-2 inhibitors (SGLT2i) head-to-head against other glucose-lowering therapies, including dipeptidyl peptidase 4 inhibitor (DDP4i) or glucagon-like peptide-1 receptor agonist (GLP-1RA)—which also have cardiovascular benefits—in patients with heart failure with reduced (HFrEF) or preserved (HFpEF) ejection fraction.

**Methods:**

Medicare fee-for-service data (2013–2019) were used to create four pair-wise comparison cohorts of type 2 diabetes patients with: (1a) HFrEF initiating SGLT2i versus DPP4i; (1b) HFrEF initiating SGLT2i versus GLP-1RA; (2a) HFpEF initiating SGLT2i versus DPP4i; and (2b) HFpEF initiating SGLT2i versus GLP-1RA. The primary outcomes were (1) hospitalization for heart failure (HHF) and (2) myocardial infarction (MI) or stroke hospitalizations. Adjusted hazards ratios (HR) and 95% CIs were estimated using inverse probability of treatment weighting.

**Results:**

Among HFrEF patients, initiation of SGLT2i versus DPP4i (cohort 1a; n = 13,882) was associated with a lower risk of HHF (adjusted Hazard Ratio [HR (95% confidence interval)], 0.67 (0.63, 0.72) and MI or stroke (HR: 0.86 [0.75, 0.99]), and initiation of SGLT2i versus GLP-1RA (cohort 1b; n = 6951) was associated with lower risk of HHF (HR: 0.86 [0.79, 0.93]), but not MI or stroke (HR: 1.02 [0.85, 1.22]). Among HFpEF patients, initiation of SGLT2i versus DPP4i (cohort 2a; n = 17,493) was associated with lower risk of HHF (HR: 0.65 [0.61, 0.69]) but not MI or stroke (HR: 0.90 [0.79, 1.02]), and initiation of SGLT2i versus GLP-1RA (cohort 2b; n = 9053) was associated with lower risk of HHF (0.89 [0.83, 0.96]), but not MI or stroke (HR: 0.97 [0.83, 1.14]). Results were robust across range of secondary outcomes (e.g., all-cause mortality) and sensitivity analyses.

**Conclusions:**

Bias from residual confounding cannot be ruled out. Use of SGLT2i was associated with reduced risk of HHF against DPP4i and GLP-1RA, reduced risk of MI or stroke against DPP4i within the HFrEF subgroup, and comparable risk of MI or stroke against GLP-1RA. Notably, the magnitude of cardiovascular benefit conferred by SGLT2i was similar among patients with HFrEF and HFpEF.

**Supplementary Information:**

The online version contains supplementary material available at 10.1186/s12933-023-01784-w.

## Introduction

The epidemiological trends for the incidence and prevalence of type 2 diabetes (T2D), heart failure (HF), and their co-occurrence have continued to worsen in the US and globally [1]. Diabetes is present in nearly half of HF patients, and the prevalence of HF is estimated to be 20% among T2D patients [2]. Compared to T2D alone, the co-existence of T2D and heart failure augurs a clinical course characterized by greater insulin resistance, accelerated progression of T2DM, and an elevated risk of cardiovascular events and mortality [3, 4]. Recently, large cardiovascular outcome trials (CVOTs) have demonstrated the efficacy of a newer medication class: sodium–glucose cotransporter 2 inhibitors (SGLT2i) in reducing the incidence of hospitalizations for heart failure (HHF) and major adverse cardiovascular events (MACE)—comprised of myocardial infarction (MI), ischemic stroke, and cardiovascular death [5, 6]. In recent CVOTs that were initially conducted among patients with heart failure with reduced ejection fraction (HFrEF), and subsequently among patients with heart failure with preserved ejection fraction (HFpEF), SGLT2i reduced the incidence of HHF by approximately 30% and improved heart-failure specific endpoints such as Kansas City Cardiomyopathy Questionnaire scores and N-terminal pro b-type natriuretic peptide levels [7]. However, these trials did not assess MACE endpoints such as MI or stroke hospitalizations [8–10], which are major contributors to cardiovascular morbidity and mortality among HFrEF and HFpEF patients [11, 12].

Similar to SGLT2i, glucagon-like peptide-1 receptor agonists (GLP-1RA) are a newer medication class with demonstrated benefits on MACE (12% risk reduction against placebo) and HHF (9% risk reduction against placebo) [13], leading to some speculation that they may also exert beneficial cardiovascular effects in patients with HF [14]. Consensus recommendations exist preferencing use of SGLT2i for T2D patients with heart failure and GLP-1RA for T2D patients with atherosclerotic cardiovascular disease [15, 16], Currently, no prospective or observational study has directly compared the magnitude of cardiovascular benefits conferred by SGLT2i head-to-head against any second-line glucose lowering therapies, including GLP-1RA, in patients with either HFrEF or HFpEF.

Accordingly, in a cohort of older adults, who have the highest prevalence of T2D and HF of any age group [17, 18], this study aimed to assess the cardiovascular effectiveness of SGLT2i compared to DPP4i and GLP-1RA among patients with HFrEF and HFpEF.

## Methods

The study was approved by the Rutgers University Institutional Review Board, and the appropriate data use agreements were in place.

### Data sources

Study subjects were drawn from Medicare insurance claims, a US federal program that provides healthcare to US citizens over 65 years of age. More specifically, we utilized a 50% random sample of Medicare fee-for-service beneficiaries enrolled in Part D from March 2013—coinciding with the approval of SGLT2i in the US—to December 2019. Data elements of interest included patient demographics, medical and pharmacy monthly enrollment status, inpatient and outpatient medical service use (International Classification of Disease [ICD], Ninth and Tenth Revisions; Current Procedural Terminology codes, Fourth Edition), and outpatient pharmacy dispensing data (drug name and strength, units dispensed, and days’ supply).

### Study population and exposure definition

Within the database, a separate cohort was created for each pairwise comparison of SGLT2i versus an alternative non-gliflozin class. Cohort membership required patients to be new users of the study medications of interest (defined as no use of the medications in the 365-day washout period preceding medication initiation), be older than 65 years of age at cohort entry and have no evidence of gestational or type 1 diabetes (T1D), cancer, end-stage renal disease, or human immunodeficiency virus infection. With the sole exception of heart failure phenotype (see below), all baseline covariates including eligibility criteria and patient characteristics were assessed in the 365 days prior to the date of medication initiation.

The study cohort was further restricted to patients with the presence of HHF with ICD codes corresponding to HFrEF (ICD-9: 428.2× or ICD-10: I50.2×) or HFpEF (ICD-9: 428.3 × or ICD-10: I50.3×) in either the first or second position of the inpatient discharge diagnosis using all available lookback. The positive predictive value for this approach for identifying patients with HFrEF is 72% and 90% using ejection fraction [EF] thresholds of ≤ 40% and ≤ 50%, respectively, and 92% for HFpEF for an EF threshold of > 50% [19]. Patients with evidence of both or neither HF subtypes were excluded from analyses.

The study was comprised of four pairwise comparison cohorts, which included patients with: (1a) HFrEF initiating SGLT2i versus DPP4i; (1b) HFrEF initiating SGLT2i versus GLP-1RA; (2a) HFpEF initiating SGLT2i or DPP4i; and (2b) HFpEF initiating SGLT2i or GLP-1RA. For SGLT2i versus DPP4i comparisons, patients using combination empagliflozin–linagliptin therapy were excluded from analysis. Further, individuals initiating SGLT2i and the comparator on the same day were also excluded from analyses. Patients meeting the inclusion and exclusion criteria could contribute to each cohort only once, but the same patient could be included in more than one cohort.

### Follow-up and study end points

Separately for each study outcome, patients began contributing to follow-up time on the day after cohort entry (i.e., medication initiation) up until the first occurrence of one of the following: end of pharmacy or health care eligibility, medication discontinuation defined as 60-day gap in treatment, medication switching (e.g., patients in SGLT2i arm initiating non-gliflozin therapy and vice versa), end of study data (December 2019), or the occurrence of the outcome.

The two primary outcomes of interest were (1) hospitalization for heart failure (HHF) (positive predictive value [PPV]: > 90%) [20], and (2) MI (PPV = 94%) or stroke (PPV = 85%) hospitalizations [21, 22]. Analysis for each of the two primary outcomes was conducted independently of the other.

### Baseline covariates and inverse probability of treatment weighting

To mitigate risk of confounding, we assessed and adjusted for > 30 baseline covariates that were assessed in the 12-month period prior to and including the index date. These covariates included patient sociodemographics (e.g., age at medication initiation, biological sex, and race, calendar year), complications of diabetes (e.g., diabetic neuropathy, nephropathy, retinopathy), oral and injectable glucose lowering therapies (e.g., metformin, sulfonylureas, insulin), diagnosis of cardiovascular conditions (e.g., myocardial infarction, stroke, HF), and cardiovascular medication use (e.g., dispensing of β-blockers, loop diuretics, statins). Frailty status was ascertained using the claims based frailty index, and using a threshold of ≥ 0.25 to define frailty [23].

Propensity scores were estimated using a logistic regression that modelled the probability of initiating SGLT2i (exposure) versus a non-gliflozin medication (control) conditional on the baseline covariates. These propensity scores were then used to estimate stabilized inverse probability of treatment weights (IPTW) to account for imbalances in patient characteristics [24].

### Statistical analysis

We assessed the performance of propensity scores based IPTW to control for confounding by examining the distribution of baseline covariates prior and after IPT weighting, and using a threshold of 10% in standardized difference as a metric for a meaningful imbalance [25]. Using an as-treated approach, where patients were censored on treatment discontinuation or switching, we estimated the rates of the primary outcomes among patients using SGLT2i (exposure) or non-gliflozin medications (control) by calculating the number of events and incidence rates (IRs). Adjusted incidence-rate differences (RD) and hazard ratios (HR) along with their 95% confidence intervals (CIs) were modelled through weighted Cox and Poisson regressions respectively.

Sensitivity and secondary analyses were conducted to assess the robustness of the study findings. First, we examined several secondary outcomes including a composite of the two primary outcomes (i.e., HF, MI or stroke hospitalizations), as well as individually examined MI hospitalizations, stroke hospitalizations, and all-cause mortality. Second, we conducted sensitivity analyses varying exposure-related censoring criteria, where instead of censoring patients at the time of treatment switching or discontinuation, we carried the index exposure forward to mimic an intention-to-treat approach with a maximum follow up truncated to 2 years.

Third, as our primary definitions to identify HF subtypes prioritize positive predictive values at the possible cost of lowered sensitivity (i.e., under-detection of patients with HF), we also employed alternative-more sensitive-HF definitions to identify HFrEF and HFpEF patients. More specifically, we allowed patients to be included in the study if they had presence of relevant HF codes in (1) any position of the inpatient discharge diagnosis, or (2) any inpatient or outpatient diagnoses fields. Fourth, we conducted sensitivity analyses where we excluded patients with a recent hospitalization (i.e., 30-days prior to the index date). Finally, to assess impact of the study estimates across calendar time, we also estimated stratified results before and after 2016. Other eligibility criteria (e.g., no evidence of T1D) were similar for all cohorts. For all cohorts, pairwise comparisons, and sensitivity analyses, the propensity scores were re-estimated, and stabilized inverse probability of treatment weights were re-calculated. All analyses were performed using SAS 9.4 (SAS Institute Inc, Cary, NC).

## Results

Within the data, we identified 240,252 patients who had evidence of heart failure, type 2 diabetes, and had used glucose lowering therapies (see Additional file 1: Appendix Fig. S1 for the CONSORT flow diagram). After the criteria of new use were applied, there were 23,959 and 95,564 remaining initiators of SGLT2i and DPP4i, respectively, and 27,340 and 35,111 eligible initiations for SGLT2i and GLP-1RA, respectively. After requirements for pertinent heart failure hospitalizations and other exclusion criteria (e.g., T1D) were applied, the four pair-wise comparison cohorts were comprised of: (Cohort 1a) 13,882 HFrEF patients initiating SGLT2i versus DPP4i; (1b) 6951 HFrEF patients initiating SGLT2i versus GLP-1RA; (2a) 17,493 HFpEF patients initiating SGLT2i versus DPP4i; and (2b) 9053 HFpEF patients initiating SGLT2i versus GLP-1RA (see Additional file 1: Appendix Tables S1–S4 for information on unadjusted and adjusted baseline characteristics for all four cohorts).

Prior to IPT weighting, SGLT2i users differed from their non-gliflozin counterparts with respect to pertinent baseline characteristics (defined as a standardized difference > 10%). Regardless of HF subtype, patients initiating DPP4i (compared to SGLT2i) were older and more likely to be diagnosed with MI, peripheral vascular disease and renal insufficiency, while those initiating GLP-1RA (compared to SGLT2i) were more likely to be female, have previously used insulin, and have evidence of microvascular complications of diabetes.

After IPT weighting, baseline characteristics in all four cohorts were well balanced with no standardized difference exceeding 10% (Tables 1 and 2). The most commonly observed guideline-directed medical therapy for HFrEF was beta blockers, followed by ACE inhibitors or ARBs, while approximately one-third of the patients were on aldosterone antagonists. Reasons for censoring were similar among the four study cohorts, and discontinuation of index exposure was the most prevalent contributor to censoring (Additional file 1: Appendix Table S5).

**Table 1:** Baseline characteristics after IPT weighting among patients with heart failure with reduced ejection fraction

	SGLT2i versus DPP4i		SD^b^	SGLT2i versus GLP-1RA		SD^b^
	SGLT2i	DPP4i		SGLT2i	GLP-1RA	
	(n = 2503)	(n = 2503)^a^		(n = 3214)	(n = 3214)^a^	
Sociodemographics						
Age, mean (SD)	73.3 (10.1)	73.8 (11.1)	3.3	69.9 (10.9)	69.9 (10.6)	0.0
Male	1326 (54.8)	1438 (57.2)	4.9	1973 (61.1)	1955 (61.0)	0.2
Race, White	1754 (72.5)	1795 (71.4)	2.3	2372 (73.5)	2350 (73.3)	0.3
Race, Black	357 (14.8)	385 (15.3)	1.6	454 (14.1)	448 (14.0)	0.2
Other Race	309 (12.8)	332 (13.2)	1.4	402 (12.5)	407 (12.7)	0.7
Calendar year (2013, 2014, 2015)	831 (34.3)	895 (35.6)	2.8	601 (18.6)	575 (17.9)	1.7
Calendar year (2016, 2017)	753 (31.1)	775 (30.9)	0.6	961 (29.8)	960 (30.0)	0.4
Calendar year (2018, 2019)	837 (34.6)	842 (33.5)	2.2	1666 (51.6)	1670 (52.1)	1.0
Diabetes-related factors						
Metformin	1240 (51.2)	1247 (49.6)	3.1	1586 (49.1)	1582 (49.4)	0.5
Sulfonylureas	1034 (42.7)	1026 (40.8)	3.9	1273 (39.4)	1265 (39.5)	0.1
DPP4i				962 (29.8)	946 (29.5)	0.6
GLP-1RA	119 (4.9)	122 (4.9)	0.1			
Insulin	850 (35.1)	833 (33.2)	4.2	1698 (52.6)	1685 (52.6)	0.1
Thiazolidinediones	105 (4.3)	110 (4.4)	0.1	145 (4.5)	146 (4.5)	0.2
Diabetes, ocular complications	343 (14.2)	377 (15.0)	2.4	624 (19.3)	630 (19.7)	0.8
Diabetes, renal complications	905 (37.4)	950 (37.8)	0.8	1326 (41.1)	1336 (41.7)	1.2
Diabetes, neurological complications	925 (38.2)	928 (36.9)	2.6	1444 (44.7)	1430 (44.6)	0.2
Other factors						
Frailty status	612 (25.3)	554 (22.1)	7.6	446 (13.8)	435 (13.6)	0.7
Myocardial infarction	177 (7.3)	214 (8.5)	4.4	189 (5.9)	181 (5.6)	0.9
Stroke	46 (1.9)	44 (1.8)	1.1	38 (1.2)	38 (1.2)	0.1
Peripheral vascular disease	832 (34.4)	833 (33.2)	2.6	951 (29.5)	938 (29.3)	0.5
Other ischemic heart disease	2008 (83.0)	2100 (83.6)	1.5	2633 (81.6)	2615 (81.6)	0.0
Renal insufficiency	1454 (60.1)	1519 (60.5)	0.8	1713 (53.1)	1711 (53.4)	0.6
ACE inhibitors	1226 (50.6)	1272 (50.6)	0.1	1572 (48.7)	1553 (48.5)	0.5
ARBs	674 (27.8)	693 (27.6)	0.6	936 (29.0)	926 (28.9)	0.2
Beta blockers	2163 (89.4)	2245 (89.3)	0.1	2887 (89.5)	2868 (89.5)	0.1
Calcium channel blockers	528 (21.8)	606 (24.1)	5.4	676 (21.0)	675 (21.1)	0.3
Non-dihydropyridine CCB	226 (9.3)	224 (8.9)	1.5	238 (7.4)	236 (7.4)	0.1
Thiazide diuretics	538 (22.2)	516 (20.5)	4.1	633 (19.6)	636 (19.8)	0.6
Loop diuretics	2041 (84.3)	2118 (84.3)	0.1	2687 (83.3)	2663 (83.1)	0.4
Aldosterone antagonists	745 (30.8)	799 (31.8)	2.2	1128 (34.9)	1119 (34.9)	0.1
Digoxin	486 (20.1)	497 (19.8)	0.7	559 (17.3)	557 (17.4)	0.2
Hydralazine/isosorbide	698 (28.8)	692 (27.5)	2.9	829 (25.7)	835 (26.1)	0.9
Other HF medications^c^	141 (5.8)	139 (5.5)	1.2	320 (9.9)	318 (9.9)	0.0
Statins	1919 (79.3)	2020 (80.4)	2.7	2711 (84.0)	2698 (84.2)	0.5
Anticoagulants	1018 (42.1)	1069 (42.6)	1.0	1361 (42.2)	1346 (42.0)	0.3
Antiplatelets	833 (34.4)	824 (32.8)	3.4	1113 (34.5)	1112 (34.7)	0.4

SGLT2i: sodium–glucose cotransporter-2 inhibitor; DPP4i: Dipeptidyl peptidase 4 inhibitor; SD: standardized difference; IPT: Inverse probability of treatment; ACE: Angiotensin converting enzyme; ARB: Angiotensin II receptor blockers; CCB: calcium channel blocker; StD: Standard Deviation; HF: Heart Failure; GLP-1RA: glucagon-like peptide 1 receptor agonist

^a^For ease of interpretation, the denominator of the DPP4i and GLP-1RA arms are weighted down to the SGLT2i group

^b^Standardized differences greater than 10% imply a meaningful difference in the patient characteristic. After IPTW weighting, there were no differences that exceeded this threshold

^c^Include angiotensin receptor-neprilysin Inhibitors and hyperpolarization-activated cyclic nucleotide-gated (HCN) channel blockers

**Table 2:** Baseline characteristics after IPT weighting among patients with heart failure with preserved ejection fraction

	SGLT2i versus DPP4i		SD^b^	SGLT2i versus GLP-1RA		SD^b^
	SGLT2i	DPP4i		SGLT2i	GLP-1RA	
	(n = 2846)	(n = 2846)^a^		(n = 3578)	(n = 3578)^a^	
Sociodemographics						
Age, mean (SD)	75.7 (10.1)	76.0 (11.0)	2.0	71.3 (10.9)	71.4 (10.5)	0.6
Male	1030 (36.9)	1069 (37.4)	0.9	1489 (41.4)	1487 (41.6)	0.5
Race, White	2039 (73.2)	2108 (73.7)	1.2	2692 (74.8)	2667 (74.7)	0.4
Race, Black	403 (14.5)	396 (13.8)	1.8	461 (12.8)	459 (12.8)	0.1
Other Race	345 (12.4)	356 (12.5)	0.3	445 (12.4)	446 (12.5)	0.4
Calendar year (2013, 2014, 2015)	894 (32.1)	939 (32.8)	1.6	696 (19.4)	669 (18.7)	1.6
Calendar year (2016, 2017)	871 (31.2)	896 (31.3)	0.2	1089 (30.3)	1082 (30.3)	0.1
Calendar year (2018, 2019)	1023 (36.7)	1026 (35.9)	1.7	1813 (50.4)	1820 (51.0)	1.2
Diabetes-related factors						
Metformin	1375 (49.3)	1378 (48.2)	2.3	1735 (48.2)	1714 (48.0)	0.4
Sulfonylureas	1094 (39.2)	1119 (39.1)	0.2	1356 (37.7)	1328 (37.2)	1.0
DPP4i				1033 (28.7)	1028 (28.8)	0.2
GLP-1RA	154 (5.5)	160 (5.6)	0.3			
Insulin	998 (35.8)	1026 (35.9)	0.2	2034 (56.5)	2003 (56.1)	0.9
Thiazolidinediones	161 (5.8)	165 (5.8)	0.1	217 (6.0)	212 (5.9)	0.3
Diabetes, ocular complications	433 (15.5)	447 (15.6)	0.3	736 (20.5)	743 (20.8)	0.8
Diabetes, renal complications	1144 (41.0)	1164 (40.7)	0.7	1605 (44.6)	1608 (45.0)	0.8
Diabetes, neurological complications	1209 (43.4)	1197 (41.9)	3.1	1851 (51.4)	1824 (51.1)	0.7
Cardiovascular factors						
Frailty status	800 (28.7)	777 (27.1)	3.4	683 (19.0)	676 (18.9)	0.1
Myocardial infarction	216 (7.8)	241 (8.4)	2.4	202 (5.6)	210 (5.9)	1.1
Stroke	54 (1.9)	59 (2.1)	0.9	53 (1.5)	56 (1.6)	0.8
Peripheral vascular disease	995 (35.7)	972 (34.0)	3.6	1103 (30.6)	1103 (30.9)	0.5
Other ischemic heart disease	1905 (68.3)	1985 (69.4)	2.3	2410 (67.0)	2422 (67.8)	1.8
Renal insufficiency	1753 (62.9)	1785 (62.4)	1.0	2052 (57.0)	2052 (57.5)	0.9
ACE inhibitors	1101 (39.5)	1103 (38.6)	1.9	1424 (39.6)	1400 (39.2)	0.8
ARBs	754 (27.0)	821 (28.7)	3.7	1107 (30.8)	1088 (30.5)	0.6
Beta blockers	2181 (78.3)	2249 (78.6)	1.0	2759 (76.7)	2763 (77.3)	1.6
Calcium channel blockers	1040 (37.3)	1097 (38.3)	2.1	1269 (35.3)	1268 (35.5)	0.5
Non-dihydropyridine CCB	488 (17.5)	482 (16.9)	1.7	510 (14.2)	507 (14.2)	0.0
Thiazide diuretics	701 (25.2)	694 (24.3)	2.0	930 (25.8)	916 (25.6)	0.5
Loop diuretics	2351 (84.3)	2386 (83.4)	2.5	3017 (83.8)	2994 (83.8)	0.0
Aldosterone antagonists	550 (19.7)	562 (19.7)	0.1	787 (21.9)	773 (21.6)	0.6
Digoxin	315 (11.3)	335 (11.7)	1.2	309 (8.6)	313 (8.8)	0.6
Hydralazine/isosorbide	764 (27.4)	800 (28.0)	1.3	928 (25.8)	939 (26.3)	1.1
Other HF medications^c^	24 (0.8)	25 (0.9)	0.4	51 (1.4)	51 (1.4)	0.0
Statins	2065 (74.1)	2166 (75.7)	3.7	2869 (79.7)	2855 (79.9)	0.5
Anticoagulants	1187 (42.6)	1170 (40.9)	3.4	1402 (38.9)	1408 (39.4)	1.0
Antiplatelets	695 (24.9)	715 (25.0)	0.1	921 (25.6)	924 (25.9)	0.6

SGLT2i: sodium–glucose cotransporter-2 inhibitor; DPP4i: Dipeptidyl peptidase 4 inhibitor; SD: standardized difference; IPT: Inverse probability of treatment; ACE: Angiotensin converting enzyme; ARB: Angiotensin II receptor blockers; CCB: calcium channel blocker; StD: Standard Deviation; HF: Heart Failure; GLP-1RA: glucagon-like peptide 1 receptor agonist

^a^For ease of interpretation, the denominator of the DPP4i and GLP-1RA arms are weighted down to the SGLT2i group

^b^Standardized differences are expressed in percentage points. Values greater than 10% imply a meaningful difference in the patient characteristic. After IPTW weighting, there were no differences that exceeded this threshold

^c^Include angiotensin receptor-neprilysin Inhibitors and hyperpolarization-activated cyclic nucleotide-gated (HCN) channel blockers

### HFrEF analysis

For the SGLT2i versus DPP4i comparison (Cohort 1a) and over a median follow up of 6.5–7.1 months, there were 704 (incidence rate per 100 person-year [IR] = 52.2) HHF events in the SGLT2i group compared to 5567 (IR = 80.9) events in the DPP4i group (Table 3), and 144 (IR = 8.8) versus 1240 (IR = 12.7) MI or stroke events for SGLT2i versus DPP4i users. After adjustment, SGLT2i users had a lower risk of both HHF: HR 0.67 (95% CI 0.63, 0.72), and MI or stroke: HR 0.86 (95% CI 0.75, 0.99). This corresponded to an adjusted rate difference [RD] per 100 person-year of − 20.9 (95% CI − 16.1, − 25.7) and − 1.6 (95% CI − 3.7, − 0.0) for the outcomes of HHF and MI or stroke, respectively.

**Table 3:** Risk of cardiovascular hospitalizations among patients initiating SGLT2i compared to non-gliflozin therapies, by heart failure subtype

HFrEF	Unadjusted		IPTW adjusted	IPTW adjusted HR (95% CI)
	No. events (IR)^a^		RD (95% CI)	
	SGLT2i	DPP4i		
	(n = 2503)	(n = 11,379)		
Follow up, months: median (IQR)	6.5 (5.1, 12.9)	7.1 (4.0, 14.3)		
Heart failure hospitalizations	704 (52.2)	5567 (80.9)	− 20.9 (− 16.1, − 25.7)	0.67 (0.63, 0.72)
MI or Stroke hospitalizations	144 (8.8)	1240 (12.7)	− 1.6 (− 3.7, − 0.0)	0.86 (0.75, 0.99)

	SGLT2i	GLP-1RA		
	(n = 3214)	(n = 3737)		
Follow up, median (IQR)	6.6 (4.3, 14.7)	7.3 (4.1, 15.1)		
Heart failure hospitalizations	893 (49.4)	1316 (60.6)	− 5.5 (− 10.0, − 1.0)	0.86 (0.79, 0.93)
MI or Stroke hospitalizations	180 (8.4)	282 (10.2)	0.6 (− 1.4, 2.5)	1.02 (0.85, 1.22)

HFpEF	Unadjusted		IPTW adjusted	IPTW adjusted HR (95% CI)
	No. events (IR)^a^		RD (95% CI)	
	SGLT2i	DPP4i		
	(n = 2846)	(n = 14,647)		
Follow up, median (IQR)	6.1 (4.0, 11.2)	6.6 (4.0, 12.5)		
Heart failure hospitalizations	804 (51.2)	7132 (80.5)	− 23.0 (− 14.5, − 31.4)	0.65 (0.61, 0.69)
MI or Stroke hospitalizations	167 (8.8)	1624 (12.7)	− 1.0 (− 3.0, 1.1)	0.90 (0.79, 1.02)

	SGLT2i	GLP-1RA		
	(n = 3578)	(n = 5475)		
Follow up, median (IQR)	6.5 (4.1, 12.8)	7.1 (4.1, 13.6)		
Heart failure hospitalizations	1059 (53.2)	2004 (60.2)	− 4.7 (− 8.6, − 0.7)	0.89 (0.83, 0.96)
MI or Stroke hospitalizations	213 (8.8)	443 (10.3)	− 0.4 (− 2.1, 1.3)	0.97 (0.83, 1.14)

HFrEF: Heart failure with reduced ejection fraction; HFpEF: Heart failure with preserved ejection fraction; SGLT2i: sodium–glucose cotransporter-2 inhibitors; DPP4i: dipeptidyl peptidase 4 inhibitors; GLP-1RA: Glucagon-like peptide-1 receptor agonists; CI: confidence intervals; IR: incidence rate; HR: hazard ratio; IQR: Interquartile range; RD: Rate difference

^a^Represent the unadjusted number of events and incidence rates per 100 person-years of follow up

^b^Hazard ratios were adjusted for variables described in Tables 1 and 2 using stabilized inverse probability of treatment weighting

Meanwhile, for the SGLT2i versus GLP-1RA comparison (Cohort 1b), over a median follow up of 6.6–7.3 months, there were 893 (IR = 49.4) versus 1316 (IR = 60.6) HHF events, and 180 (IR = 8.4) versus 282 (IR = 10.2) MI or stroke events. After adjustment, SGLT2i use was associated with a lower risk of HHF: HR 0.86 (95% CI 0.79, 0.93) and RD − 5.5 (95% CI − 10.0,-1.0), but not MI or stroke, HR 1.02 (95% CI 0.85, 1.22) and RD 0.6 (95% CI − 1.4, 2.5).

### HFpEF analysis

For the SGLT2i versus DPP4i comparison (Cohort 2a), over a median follow up of 6.1–6.6 months, there were 804 (IR = 51.2) versus 7132 (IR = 80.5) HHF events, and 167 (IR = 8.8) versus 1624 (IR = 12.7) MI or stroke events (Table 3). After adjustment, SGLT2i users exhibited significant reductions in risk of HHF: HR 0.65 (95% CI 0.61, 0.69) and RD − 23.0 (95% CI − 14.5, − 31.4), and numerical decreases in MI or stroke that did not reach statistical significance: HR 0.90 (95% CI 0.79, 1.02) and RD − 1.0 (95% CI − 3.0, 1.1).

For the SGLT2i versus GLP-1RA comparison (Cohort 2b), over a median follow up of 6.5–7.1 months, there were 1059 (IR = 53.2) versus 2004 (IR = 60.2) HHF events, and 213 (8.8) versus 443 (10.3) MI or stroke events. After adjustment, SGLT2i use was associated with a lower risk of HHF: HR 0.89 (95% CI 0.83, 0.96) and RD − 4.7 (95% CI − 8.6, − 0.7), but not MI or stroke, HR 0.97 (95% CI 0.83, 1.14) and RD − 0.4 (− 2.1, 1.3).

Notably, the magnitude of reduction in cardiovascular outcomes conferred by SGLT2i appeared to be similar for both the HFrEF and HFpEF cohorts with no evidence of interaction, and all p-values for heterogeneity were > 0.05 for all hazard ratios and rate differences.

### Sensitivity and secondary analysis

SGLT2i use was associated with a reduced risk for the (1) endpoint comprised of MI, stroke or HF hospitalizations, and (2) all-cause mortality against DPP4i regardless of HF subtype and was associated with a significant reduction in MI hospitalizations in the HFrEF but not the HFpEF cohort (Table 4). However, SGLT2i and GLP-1RA were similar in terms of all non-HF related endpoints. Findings for the sensitivity analysis using an intention-to-treat approach were consistent with the primary analyses though closer to null, and non-statistically significant for MI or stroke hospitalizations for any HF subtype.

**Table 4:** Adjusted risk of cardiovascular outcomes among patients initiating SGLT2i compared to non-gliflozin therapies, by heart failure subtype: Sensitivity and secondary analysis

*HFrEF*	SGLT2i versus DPP4i	SGLT2i versus GLP-1RA
Other secondary outcomes		
MI hospitalizations	0.81 (0.69, 0.95)	1.01 (0.83, 1.23)
Stroke hospitalizations	1.02 (0.74, 1.41)	0.97 (0.63, 1.50)
All-cause mortality	0.39 (0.34, 0.46)	0.86 (0.72, 1.03)
MI, stroke or HF hospitalizations	0.88 (0.82, 0.95)	0.98 (0.90, 1.06)
Intention to treat analyses		
HF hospitalizations	0.79 (0.74, 0.83)	0.89 (0.84, 0.95)
MI or stroke hospitalizations	1.09 (1.00, 1.20)	0.99 (0.88, 1.12)

*HFpEF*	SGLT2i versus DPP4i	SGLT2i versus GLP-1RA
Other secondary outcomes		
MI hospitalizations	0.88 (0.76, 1.02)	0.96 (0.80, 1.14)
Stroke hospitalizations	0.90 (0.68, 1.21)	0.94 (0.67, 1.33)
All-cause mortality	0.46 (0.40, 0.52)	0.94 (0.80, 1.10)
MI, stroke or HF hospitalizations	0.91 (0.85, 0.97)	0.96 (0.89, 1.03)
Intention to treat analyses		
HF hospitalizations	0.76 (0.72, 0.80)	0.91 (0.86, 0.97)
MI or stroke hospitalizations	1.00 (0.92, 1.11)	1.00 (0.90, 1.11)

SGLT2i: sodium–glucose cotransporter-2 inhibitors; DPP4i: dipeptidyl peptidase 4 inhibitors; GLP-1RA: Glucagon-like peptide-1 receptor agonists; CI: confidence intervals; IR: incidence rate; HR: hazard ratio

Hazard ratios adjusted for variables described in Table 1 and 2 using stabilized inverse probability of treatment weighting

See Additional file 1: Appendix Table S6 for number of events and incidence rates for secondary outcomes and sensitivity analysis

Study results were robust to alternative definitions for identifying heart failure phenotypes for HHF (Table 5). For the MI or stroke outcome, while the point estimates were similar to the primary analysis, only two alternative HF definitions for HFpEF yielded statistical significance against DPP4i whereas all others crossed the null. Outcomes were robust when stratified by calendar year—albeit underpowered, and accordingly MI or stroke hospitalization risk for Cohort 2a was not significantly different following 2016.

**Table 5:** Adjusted risk of cardiovascular outcomes among patients initiating SGLT2i compared to non-gliflozin therapies, using alternative definitions for HF subtypes

*HFrEF*	SGLT2i versus DPP4i	SGLT2i versus GLP-1RA
HF alternative definition 1^a^		
HF hospitalizations	0.68 (0.64, 0.72)	0.84 (0.79, 0.91)
MI or stroke hospitalizations	0.92 (0.83, 1.03)	1.01 (0.87, 1.11)
HF alternative definition 2^a^		
HF hospitalizations	0.68 (0.64, 0.71)	0.85 (0.80, 0.90)
MI or stroke hospitalizations	0.91 (0.82, 1.01)	1.00 (0.87, 1.14)
HF alternative definition 3^a^		
HF hospitalizations	0.71 (0.66, 0.77)	0.86 (0.79, 0.94)
MI or stroke hospitalizations	0.88 (0.75, 1.03)	1.04 (0.86, 1.26)
Year ≤ 2016		
HF hospitalizations	0.67 (0.59, 0.77)	0.82 (0.71, 0.94)
MI or stroke hospitalizations	0.70 (0.53, 0.94)	1.00 (0.75, 1.34)
Year > 2016		
HF hospitalizations	0.61 (0.55, 0.67)	0.84 (0.76, 0.92)
MI or stroke hospitalizations	0.93 (0.77, 1.11)	0.93 (0.76, 1.22)

*HFpEF*	SGLT2i versus DPP4i	SGLT2i versus GLP-1RA
HF alternative definition 1^a^		
HF hospitalizations	0.63 (0.60, 0.67)	0.85 (0.80, 0.91)
MI or stroke hospitalizations	0.88 (0.79, 0.98)	0.97 (0.85, 1.10)
HF alternative definition 2^a^		
HF hospitalizations	0.63 (0.60, 0.66)	0.83 (0.79, 0.88)
MI or stroke hospitalizations	0.90 (0.81, 0.99)	0.96 (0.85, 1.08)
HF alternative definition 3^a^		
HF hospitalizations	0.66 (0.62, 0.72)	0.88 (0.81, 0.95)
MI or stroke hospitalizations	0.90 (0.78, 1.03)	0.98 (0.83, 1.17)
Year ≤ 2016		
HF hospitalizations	0.70 (0.63, 0.79)	0.91 (0.83, 1.05)
MI or stroke hospitalizations	0.89 (0.71, 1.12)	1.05 (0.81, 1.35)
Year > 2016		
HF hospitalizations	0.59 (0.54, 0.64)	0.84 (0.76, 0.92)
MI or stroke hospitalizations	0.89 (0.75, 1.05)	0.93 (0.76, 1.13)

SGLT2i: sodium–glucose cotransporter-2 inhibitors; DPP4i: dipeptidyl peptidase 4 inhibitors; GLP-1RA: Glucagon-like peptide-1 receptor agonists; CI: confidence intervals; IR: incidence rate; HR: hazard ratio

Hazard ratios adjusted for variables described in Table 1 using stabilized inverse probability of treatment weighting

^a^For the primary analysis, the cohort was restricted to patients with codes corresponding to HFrEF or HFpEF in either the first or second position in the discharge diagnosis. We conducted sensitivity analysis where we reconstructed the cohort to patients with the relevant diagnoses at any inpatient discharge diagnosis (alternative definition 1), or any inpatient or outpatient diagnosis (alternative definition 2). Finally, in the third sensitivity analysis, we excluded patients with a recent HF hospitalization within 30 days of index (alternative definition 3). For all analysis, no patient could have diagnosis of HFrEF and HFpEF at the same time, and IPTW was recalculated for each cohort

## Discussion

Initial CVOTs demonstrated the cardiovascular benefits of SGLT2i against placebo on the incidence of HHF and MACE; however, the proportion of patients with concomitant type 2 diabetes and heart failure varied substantially with no distinction made between HFrEF and HFpEF. Subsequent trials dedicated solely to HFrEF and HFpEF populations found similar reductions in HHF but did not assess incidence of MACE. In this population-based cohort study comprised of older adults co-diagnosed with T2D and HF, use of SGLT2i compared to DPP4i was associated with a 33–35% and 10–14% lower risk of HHF and MI or stroke respectively, and a 11–14% lower risk of HHF and a similar risk of MI or stroke against GLP-1RA. Notably, the magnitude of cardiovascular reduction attributable to SGLT2i was comparable in both HFrEF and HFpEF cohorts.

This investigation has pertinent clinical implications. As cardiovascular events remain the primary cause of excess mortality among patients with T2D and HF [26, 27], therapeutic strategies that inform and reduce the incidence of such events can be useful in guiding patient care. Moreover, in contrast to CVOTs which compared SGLT2i against placebo, our study represents the first comprehensive effort to evaluate SGLT2i against DPP4i and more importantly, GLP-1RA. Despite their relevance to clinical medicine, such head-to-head comparison data are unlikely to be generated from clinical trials. Finally, our study findings reinforce the effectiveness of SGLT2i in reducing HHF in HFpEF patients, a condition for which few viable treatment modalities exist [28].

Our finding of SGLT2i reducing HHF is in line with previous trials and observational studies, as well as clinical guidelines that advocate their use in patients with HF [29, 30]. Comparatively, the reduction in HHF attributable to SGLT2i was less pronounced against GLP-1RA relative to DPP4i. In contrast to DPP4i—which have a neutral effect on HHF, CVOTs have shown that GLP-1RA modestly reduce the incidence of HHF between 9 and 11 percent against placebo [13]. Lastly, these findings are also in line with a recent observational study that compared SGLT2i to GLP-1RA and found a 30% reduction in HHF risk among individuals with established cardiovascular disease [31].

In contrast to their robust data for HHF, the evidence for SGLT2i is less consistent for MACE endpoints, with clinical trials indicating that such benefits are confined to patients with established cardiovascular disease. In this context, our finding of SGLT2i reducing risk of MI or stroke compared to DPP4i is consistent with earlier CVOTs where SGLT2i was evaluated against placebo. Notably, our study also demonstrated that the incidence of MI or stroke was comparable among patients initiating SGLT2i versus GLP-1RA—which have salutary effects on MACE endpoints [15, 32].

Despite their documented benefits on cardiovascular endpoints, there may exist some barriers associated with SGLT2i use among patients with HF. First, as all SGLT2i products are currently branded, high prescription drug costs may impose financial constraints among this population—which already has high levels of polypharmacy, and consequently medication-related costs [33]. Secondly, clinicians and patients may be hesitant to use these agents due to their unique adverse reaction profile that encompasses lower limb amputations, diabetic ketoacidosis and urogenital infections [34–37]; however, data from clinical trials suggests that such events do not seem to occur with greater frequency among patients with heart failure [38].

This study took several steps to mitigate concerns for confounding by restricting analysis to new users of study medications and adjusting for pertinent covariates. Patients were sourced from routine clinical care ensuring widespread generalizability of study findings to older adults. Moreover, study estimates were consistent across a range of sensitivity, secondary, and subgroup analyses. Finally, information on medication dispensing, rather than prescribing data, were available for Medicare data mitigating some concerns for exposure misclassification. However, study findings should be viewed in light of limitations. First, owing to the observational nature of the study, findings are susceptible to residual confounding. For instance, although we assessed and adjusted for several relevant confounders, information on important variables such as hemoglobin A1c, body weight or severity of HF were not directly available in Medicare data; however, prior studies have shown that balance on many of these unmeasured characteristics can be achieved with the use of claims-based proxies [39]. Second, study findings are most generalizable to older adults enrolled in Medicare fee-for-service plans. However, we would not expect the biological effects of SGLT2i to vary by insurance status. Third, our study lacked sufficient power to explore cardiovascular outcomes for individual SGLT2i. Further, given the time frame over which our study was conducted, we were unable to include more recently approved agents such as ertugliflozin or semaglutide. Finally, we were unable to study heart failure patients without diabetes as the use of SGLT2i among this population remained very low (< 0.7%) over the study period, which preceded the publication of the more recent SGLT2i trials dedicated to heart failure populations.

In conclusion, this population-based analyses found that the initiation of SGLT2i was associated with a reduced risk of HHF compared to DPP4i and GLP-1RA, reduced risk of MI or stroke compared to DPP4i, and comparable risk of MI or stroke compared to GLP-1RA. Notably, the cardiovascular benefit profile was similar in magnitude for SGLT2i across patients with HFrEF and HFpEF. These findings have important implications in prevention of cardiovascular morbidity and mortality among patients dually diagnosed with diabetes and heart failure.

## Supplementary Information

**Additional file 1. Fig. S1**: CONSORT flow diagram. **Appendix Table S1**: Baseline patient characteristics prior to after IPT weighting, SGLT2i v DPP4i among patients with heart failure with reduced ejection fraction. **Appendix Table S2**: Baseline patient characteristics prior to after IPT weighting, SGLT2i v DPP4i among patients with heart failure with preserved ejection fraction. **Appendix Table S3**: Baseline patient characteristics prior to after IPT weighting, SGLT2i v GLP-1RA among patients with heart failure with reduced ejection fraction. **Appendix Table S4**: Baseline patient characteristics prior to after IPT weighting, SGLT2i v GLP-1RA among patients with heart failure with preserved ejection fraction. **Appendix Table S5**: Follow up and reasons for censoring. **Appendix Table S6**: Risk of cardiovascular outcomes among patients initiating SGLT2i compared to other therapies, by heart failure subtype: Sensitivity and secondary analysis.

## Data Availability

The data that support the findings of this study are available from Medicare but restrictions apply to the availability of these data, which were used under license for the current study, and so are not publicly available.
