# Supplementary material for: Cardiovascular outcomes with SGLT2 inhibitors versus DPP4 inhibitors and GLP-1 receptor agonists in patients with heart failure with reduced and preserved ejection fraction
Source: Cardiovasc Diabetol. 2023 Mar 10;22:54. doi: 10.1186/s12933-023-01784-w (PMC9999503; doi:10.1186/s12933-023-01784-w)
Supplement: Supplementary file 1 — Additional file 1. Fig. S1: CONSORT flow diagram. Appendix Table S1: Baseline patient characteristics prior to after IPT weighting, SGLT2i v DPP4i among patients with heart failure with reduced ejection fraction. Appendix Table S2: Baseline patient characteristics prior to after IPT weighting, SGLT2i v DPP4i among patients with heart failure with preserved ejection fraction. Appendix Table S3: Baseline patient characteristics prior to after IPT weighting, SGLT2i v GLP-1RA among patients with heart failure with reduced ejection fraction. Appendix Table S4: Baseline patient characteristics prior to after IPT weighting, SGLT2i v GLP-1RA among patients with heart failure with preserved ejection fraction. Appendix Table S5: Follow up and reasons for censoring. Appendix Table S6: Risk of cardiovascular outcomes among patients initiating SGLT2i compared to other therapies, by heart failure subtype: Sensitivity and secondary analysis. [file 12933_2023_1784_MOESM1_ESM.docx]

**SUPPLEMENTARY APPENDIX**

**Supplementary Figure 1.** CONSORT flow diagram

**Appendix Table 1:** Baseline patient characteristics prior to after IPT weighting, SGLT2i v DPP4i among patients with heart failure with reduced ejection fraction

**Appendix Table 2:** Baseline patient characteristics prior to after IPT weighting, SGLT2i v DPP4i among patients with heart failure with preserved ejection fraction

**Appendix Table 3:** Baseline patient characteristics prior to after IPT weighting, SGLT2i v GLP-1RA among patients with heart failure with reduced ejection fraction

**Appendix Table 4:** Baseline patient characteristics prior to after IPT weighting, SGLT2i v GLP-1RA among patients with heart failure with preserved ejection fraction

**Appendix Table 5:** Follow up and reasons for censoring

**Appendix Table 6:** Risk of cardiovascular outcomes among patients initiating SGLT2i compared to other therapies, by heart failure subtype: Sensitivity and secondary analysis.

**Supplementary Figure 1. CONSORT flow table**

**
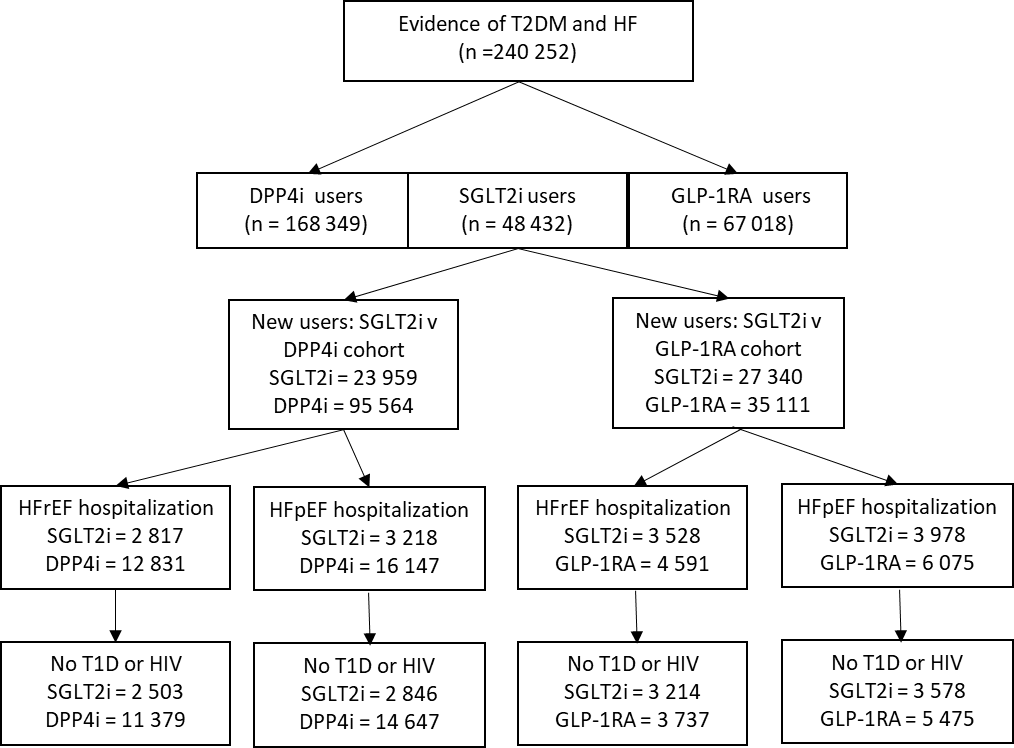
**

| **Appendix Table 1: Baseline patient characteristics prior to after IPT weighting, SGLT2i v DPP4i among patients with heart failure with reduced ejection fraction ^a^** | | | | | | |
| --- | --- | --- | --- | --- | --- | --- |
|  | **SGLT2i** | **DPP4i** | **SD ^c^** | **SGLT2i** | **DPP4i** | **SD ^c^** |
|  | **(n=2,503)** | **(n=11,379) ^b^** |  | **(n=2,503)** | **(n=2,503) ^b^** |  |
| **Sociodemographics** |  |  |  |  |  |  |
| Age, mean (Standard Deviation) | 69.3 (10.5) | 74.9 (10.8) | 37.3 | 73.3 (10.1) | 73.8 (11.1) | 3.3 |
| Male | 1,631 (65.2) | 6,296 (55.3) | 20.2 | 1,326 (54.8) | 1,438 (57.2) | 4.9 |
| Race, White | 1,876 (75.0) | 8,034 (70.6) | 9.8 | 1,754 (72.5) | 1,795 (71.4) | 2.3 |
| Race, Black | 340 (13.6) | 1,797 (15.8) | 6.2 | 357 (14.8) | 385 (15.3) | 1.6 |
| Other Race | 287 (11.5) | 1,548 (13.6) | 6.5 | 309 (12.8) | 332 (13.2) | 1.4 |
| Calendar year (2013, 2014,2015) | 348 (13.9) | 4,619 (40.6) | 62.8 | 831 (34.3) | 895 (35.6) | 2.8 |
| Calendar year (2016, 2017) | 647 (25.8) | 3,645 (32.0) | 13.7 | 753 (31.1) | 775 (30.9) | 0.6 |
| Calendar year (2018, 2019) | 1,508 (60.2) | 3,115 (27.4) | 70.2 | 837 (34.6) | 842 (33.5) | 2.2 |
| **Diabetes related factors** |  |  |  |  |  |  |
| Metformin | 1,450 (57.9) | 5,413 (47.6) | 20.9 | 1,240 (51.2) | 1,247 (49.6) | 3.1 |
| Sulfonylureas | 910 (36.4) | 4,767 (41.9) | 11.4 | 1,034 (42.7) | 1,026 (40.8) | 3.9 |
| GLP-1RA | 380 (15.2) | 265 (2.3) | 46.7 | 119 (4.9) | 122 (4.9) | 0.1 |
| Insulin | 1,159 (46.3) | 3,423 (30.1) | 33.9 | 850 (35.1) | 833 (33.2) | 4.2 |
| Thiazolidinediones | 123 (4.9) | 485 (4.3) | 3.1 | 105 (4.3) | 110 (4.4) | 0.1 |
| Diabetes, ocular complications | 451 (18.0) | 1,634 (14.4) | 9.9 | 343 (14.2) | 377 (15.0) | 2.4 |
| Diabetes, renal complications | 907 (36.2) | 4,365 (38.4) | 4.4 | 905 (37.4) | 950 (37.8) | 0.8 |
| Diabetes, neurological complications | 1,027 (41.0) | 4,042 (35.5) | 11.4 | 925 (38.2) | 928 (36.9) | 2.6 |
| **Other factors** |  |  | . |  |  | . |
| Frailty | 295 (11.8) | 2,767 (24.3) | 33.0 | 612 (25.3) | 554 (22.1) | 7.6 |
| Myocardial infarction | 116 (4.6) | 1,065 (9.4) | 18.6 | 177 (7.3) | 214 (8.5) | 4.4 |
| Stroke | 30 (1.2) | 216 (1.9) | 5.7 | 46 (1.9) | 44 (1.8) | 1.1 |
| Peripheral vascular disease | 697 (27.8) | 3,902 (34.3) | 14.0 | 832 (34.4) | 833 (33.2) | 2.6 |
| Other ischemic heart disease | 2,086 (83.3) | 9,506 (83.5) | 0.5 | 2,008 (83.0) | 2,100 (83.6) | 1.5 |
| Renal insufficiency | 1,107 (44.2) | 7,323 (64.4) | 41.3 | 1,454 (60.1) | 1,519 (60.5) | 0.8 |
| ACE inhibitors | 1,239 (49.5) | 5,793 (50.9) | 2.8 | 1,226 (50.6) | 1,272 (50.6) | 0.1 |
| ARBs | 733 (29.3) | 3,108 (27.3) | 4.4 | 674 (27.8) | 693 (27.6) | 0.6 |
| Beta blockers | 2,255 (90.1) | 10,143 (89.1) | 3.1 | 2,163 (89.4) | 2,245 (89.3) | 0.1 |
| Calcium channel blockers | 494 (19.7) | 2,879 (25.3) | 13.4 | 528 (21.8) | 606 (24.1) | 5.4 |
| Non-dihydropyridine CCB | 185 (7.4) | 1,053 (9.3) | 6.7 | 226 (9.3) | 224 (8.9) | 1.5 |
| Thiazide diuretics | 468 (18.7) | 2,392 (21.0) | 5.8 | 538 (22.2) | 516 (20.5) | 4.1 |
| Loop diuretics | 2,066 (82.5) | 9,629 (84.6) | 5.6 | 2,041 (84.3) | 2,118 (84.3) | 0.1 |
| Aldosterone antagonists | 945 (37.8) | 3,472 (30.5) | 15.3 | 745 (30.8) | 799 (31.8) | 2.2 |
| Digoxin | 459 (18.3) | 2,285 (20.1) | 4.4 | 486 (20.1) | 497 (19.8) | 0.7 |
| Hydralazine / Isosorbide | 550 (22.0) | 3,252 (28.6) | 15.2 | 698 (28.8) | 692 (27.5) | 2.9 |
| Other heart failure medications ^d^ | 346 (13.8) | 419 (3.7) | 36.5 | 141 (5.8) | 139 (5.5) | 1.2 |
| Statins | 2,125 (84.9) | 9,038 (79.4) | 14.3 | 1,919 (79.3) | 2,020 (80.4) | 2.7 |
| Anticoagulants | 1,128 (45.1) | 4,739 (41.6) | 6.9 | 1,018 (42.1) | 1,069 (42.6) | 1.0 |
| Antiplatelets | 879 (35.1) | 3,666 (32.2) | 6.1 | 833 (34.4) | 824 (32.8) | 3.4 |
| SGLT2: Sodium-glucose cotransporter-2 inhibitor; DPP4i:  Dipeptidyl peptidase 4 inhibitor; SD:  standardized difference; IPT: Inverse probability of treatment; ACE: Angiotensin converting enzyme; ARB:  Angiotensin II receptor blockers; CCB: calcium channel blocker  ^a^ Heart failure subtype was determined by requiring presence of ICD-10 codes: I50.2x or I50.3x for HFrEF and HFpEF respectively, in either the first or second position of a heart failure hospitalization. The positive predictive value for this approach exceeds 85% for identifying HFrEF and HFpEF patients.  ^b^ For ease of interpretation, the denominator of the DPP4i arm is weighted down to the SGLT2i group.  ^c^ Standardized differences greater than 10 imply a meaningful difference in the patient characteristic. After IPTW weighting, there were no differences that exceeded this threshold.  ^d^Include angiotensin receptor-neprilysin inhibitors and hyperpolarization-activated cyclic nucleotide-gated (HCN) channel blockers. | | | | | | |

| **Appendix Table 2: Baseline patient characteristics prior to after IPT weighting, SGLT2i v DPP4i among patients with heart failure with preserved ejection fraction ^a^** | | | | | | |
| --- | --- | --- | --- | --- | --- | --- |
|  | **SGLT2i** | **DPP4i** | **SD ^c^** | **SGLT2i** | **DPP4i** | **SD ^c^** |
|  | **(n=2,846)** | **(n=14,647) ^b^** |  | **(n=2,846)** | **(n=2,846) ^b^** |  |
|  |  |  |  |  |  |  |
| **Sociodemographics** |  |  |  |  |  |  |
| Age, mean (Standard Deviation) | 71.3 (10.2) | 77.0 (10.6) | 38.7 | 75.7 (10.1) | 76.0 (11.0) | 2.0 |
| Male | 1,305 (45.9) | 5,223 (35.7) | 20.9 | 1,030 (36.9) | 1,069 (37.4) | 0.9 |
| Race, White | 2,198 (77.2) | 10,680 (72.9) | 10.0 | 2,039 (73.2) | 2,108 (73.7) | 1.2 |
| Race, Black | 305 (10.7) | 2,128 (14.5) | 11.5 | 403 (14.5) | 396 (13.8) | 1.8 |
| Other Race | 343 (12.1) | 1,839 (12.6) | 1.5 | 345 (12.4) | 356 (12.5) | 0.3 |
| Calendar year (2013, 2014,2015) | 446 (15.7) | 5,323 (36.3) | 48.5 | 894 (32.1) | 939 (32.8) | 1.6 |
| Calendar year (2016, 2017) | 766 (26.9) | 4,729 (32.3) | 11.8 | 871 (31.2) | 896 (31.3) | 0.2 |
| Calendar year (2018, 2019) | 1,634 (57.4) | 4,595 (31.4) | 54.3 | 1,023 (36.7) | 1,026 (35.9) | 1.7 |
| **Diabetes related factors** |  |  | . |  |  | . |
| Metformin | 1,640 (57.6) | 6,717 (45.9) | 23.7 | 1,375 (49.3) | 1,378 (48.2) | 2.3 |
| Sulfonylureas | 984 (34.6) | 5,860 (40.0) | 11.3 | 1,094 (39.2) | 1,119 (39.1) | 0.2 |
| GLP-1RA | 519 (18.2) | 391 (2.7) | 52.6 | 154 (5.5) | 160 (5.6) | 0.3 |
| Insulin | 1,422 (50.0) | 4,832 (33.0) | 35.0 | 998 (35.8) | 1,026 (35.9) | 0.2 |
| Thiazolidinediones | 175 (6.1) | 856 (5.8) | 1.3 | 161 (5.8) | 165 (5.8) | 0.1 |
| Diabetes, ocular complications | 522 (18.3) | 2,235 (15.3) | 8.3 | 433 (15.5) | 447 (15.6) | 0.3 |
| Diabetes, renal complications | 1,059 (37.2) | 6,094 (41.6) | 9.0 | 1,144 (41.0) | 1,164 (40.7) | 0.7 |
| Diabetes, neurological complications | 1,378 (48.4) | 5,904 (40.3) | 16.4 | 1,209 (43.4) | 1,197 (41.9) | 3.1 |
| **Other factors** |  |  | . |  |  | . |
| Frailty | 450 (15.8) | 4,304 (29.4) | 32.9 | 800 (28.7) | 777 (27.1) | 3.4 |
| Myocardial infarction | 139 (4.9) | 1,343 (9.2) | 16.8 | 216 (7.8) | 241 (8.4) | 2.4 |
| Stroke | 33 (1.2) | 333 (2.3) | 8.6 | 54 (1.9) | 59 (2.1) | 0.9 |
| Peripheral vascular disease | 828 (29.1) | 5,096 (34.8) | 12.2 | 995 (35.7) | 972 (34.0) | 3.6 |
| Other ischemic heart disease | 2,016 (70.8) | 10,151 (69.3) | 3.3 | 1,905 (68.3) | 1,985 (69.4) | 2.3 |
| Renal insufficiency | 1,336 (46.9) | 9,627 (65.7) | 38.6 | 1,753 (62.9) | 1,785 (62.4) | 1.0 |
| ACE inhibitors | 1,131 (39.7) | 5,582 (38.1) | 3.3 | 1,101 (39.5) | 1,103 (38.6) | 1.9 |
| ARBs | 873 (30.7) | 4,179 (28.5) | 4.7 | 754 (27.0) | 821 (28.7) | 3.7 |
| Beta blockers | 2,235 (78.5) | 11,530 (78.7) | 0.5 | 2,181 (78.3) | 2,249 (78.6) | 1.0 |
| Calcium channel blockers | 944 (33.2) | 5,776 (39.4) | 13.1 | 1,040 (37.3) | 1,097 (38.3) | 2.1 |
| Non-dihydropyridine CCB | 424 (14.9) | 2,547 (17.4) | 6.8 | 488 (17.5) | 482 (16.9) | 1.7 |
| Thiazide diuretics | 664 (23.3) | 3,598 (24.6) | 2.9 | 701 (25.2) | 694 (24.3) | 2.0 |
| Loop diuretics | 2,331 (81.9) | 12,256 (83.7) | 4.7 | 2,351 (84.3) | 2,386 (83.4) | 2.5 |
| Aldosterone antagonists | 670 (23.5) | 2,764 (18.9) | 11.4 | 550 (19.7) | 562 (19.7) | 0.1 |
| Digoxin | 277 (9.7) | 1,774 (12.1) | 7.6 | 315 (11.3) | 335 (11.7) | 1.2 |
| Hydralazine / Isosorbide | 672 (23.6) | 4,241 (29.0) | 12.2 | 764 (27.4) | 800 (28.0) | 1.3 |
| Other heart failure medications ^d^ | 59 (2.1) | 98 (0.7) | 12.1 | 24 (0.8) | 25 (0.9) | 0.4 |
| Statins | 2,302 (80.9) | 10,954 (74.8) | 14.7 | 2,065 (74.1) | 2,166 (75.7) | 3.7 |
| Anticoagulants | 1,181 (41.5) | 5,945 (40.6) | 1.8 | 1,187 (42.6) | 1,170 (40.9) | 3.4 |
| Antiplatelets | 761 (26.7) | 3,630 (24.8) | 4.5 | 695 (24.9) | 715 (25.0) | 0.1 |
| SGLT2: Sodium-glucose cotransporter-2 inhibitor; DPP4i:  Dipeptidyl peptidase 4 inhibitor; SD:  standardized difference; IPT: Inverse probability of treatment; ACE: Angiotensin converting enzyme; ARB:  Angiotensin II receptor blockers; CCB: calcium channel blocker  ^a^ Heart failure subtype was determined by requiring presence of ICD-10 codes: I50.2x or I50.3x for HFrEF and HFpEF respectively, in either the first or second position of a heart failure hospitalization. The positive predictive value for this approach exceeds 85% for identifying HFrEF and HFpEF patients.  ^b^ For ease of interpretation, the denominator of the DPP4i arm is weighted down to the SGLT2i group.  ^c^ Standardized differences greater than 10 imply a meaningful difference in the patient characteristic. After IPTW weighting, there were no differences that exceeded this threshold.  ^d^Include angiotensin receptor-neprilysin inhibitors and hyperpolarization-activated cyclic nucleotide-gated (HCN) channel blockers. | | | | | | |

| **Appendix Table 3: Baseline patient characteristics prior to after IPT weighting, SGLT2i v GLP-1RA among patients with heart failure with reduced ejection fraction ^a^** | | | | | | |  |
| --- | --- | --- | --- | --- | --- | --- | --- |
|  | **SGLT2i** | **GLP-1RA** | **SD ^c^** | **SGLT2i** | **GLP-1RA** | **SD ^c^** |  |
|  | **(n=3,214)** | **(n=3,737) ^b^** |  | **(n=3,214)** | **(n=3,214) ^b^** |  |  |
| **Sociodemographics** |  |  |  |  |  |  |  |
| Age, mean (Standard Deviation) | 70.7 (10.5) | 69.5 (10.8) | 8.0 | 69.9 (10.9) | 69.9 (10.6) | 0.0 |  |
| Male | 2,075 (64.6) | 2,173 (58.1) | 13.2 | 1,973 (61.1) | 1,955 (61.0) | 0.2 |  |
| Race, White | 2,345 (73.0) | 2,722 (72.8) | 0.3 | 2,372 (73.5) | 2,350 (73.3) | 0.3 |  |
| Race, Black | 426 (13.3) | 555 (14.9) | 4.6 | 454 (14.1) | 448 (14.0) | 0.2 |  |
| Other Race | 443 (13.8) | 460 (12.3) | 4.4 | 402 (12.5) | 407 (12.7) | 0.7 |  |
| Calendar year (2013, 2014,2015) | 491 (15.3) | 738 (19.7) | 11.8 | 601 (18.6) | 575 (17.9) | 1.7 | |
| Calendar year (2016, 2017) | 899 (28.0) | 1,174 (31.4) | 7.5 | 961 (29.8) | 960 (30.0) | 0.4 | |
| Calendar year (2018, 2019) | 1,824 (56.8) | 1,825 (48.8) | 15.9 | 1,666 (51.6) | 1,670 (52.1) | 1.0 | |
| **Diabetes related factors** |  |  | . |  |  | . |  |
| Metformin | 1,813 (56.4) | 1,628 (43.6) | 25.9 | 1,586 (49.1) | 1,582 (49.4) | 0.5 |  |
| Sulfonylureas | 1,321 (41.1) | 1,385 (37.1) | 8.3 | 1,273 (39.4) | 1,265 (39.5) | 0.1 |  |
| GLP-1RA | 1,005 (31.3) | 1,048 (28.0) | 7.1 | 962 (29.8) | 946 (29.5) | 0.6 |  |
| Insulin | 1,240 (38.6) | 2,376 (63.6) | 51.7 | 1,698 (52.6) | 1,685 (52.6) | 0.1 |  |
| Thiazolidinediones | 177 (5.5) | 137 (3.7) | 8.8 | 145 (4.5) | 146 (4.5) | 0.2 |  |
| Diabetes, ocular complications | 539 (16.8) | 833 (22.3) | 14.0 | 624 (19.3) | 630 (19.7) | 0.8 |  |
| Diabetes, renal complications | 1,126 (35.0) | 1,795 (48.0) | 26.6 | 1,326 (41.1) | 1,336 (41.7) | 1.2 |  |
| Diabetes, neurological complications | 1,266 (39.4) | 1,813 (48.5) | 18.5 | 1,444 (44.7) | 1,430 (44.6) | 0.2 |  |
| **Other factors** |  |  | . |  |  | . |  |
| Frailty | 369 (11.5) | 566 (15.1) | 10.8 | 446 (13.8) | 435 (13.6) | 0.7 |  |
| Myocardial infarction | 146 (4.5) | 242 (6.5) | 8.5 | 189 (5.9) | 181 (5.6) | 0.9 |  |
| Stroke | 36 (1.1) | 49 (1.3) | 1.7 | 38 (1.2) | 38 (1.2) | 0.1 |  |
| Peripheral vascular disease | 894 (27.8) | 1,135 (30.4) | 5.6 | 951 (29.5) | 938 (29.3) | 0.5 |  |
| Other ischemic heart disease | 2,686 (83.6) | 2,995 (80.1) | 8.9 | 2,633 (81.6) | 2,615 (81.6) | 0.0 |  |
| Renal insufficiency | 1,424 (44.3) | 2,302 (61.6) | 35.2 | 1,713 (53.1) | 1,711 (53.4) | 0.6 |  |
| ACE inhibitors | 1,588 (49.4) | 1,766 (47.3) | 4.3 | 1,572 (48.7) | 1,553 (48.5) | 0.5 |  |
| ARBs | 987 (30.7) | 1,036 (27.7) | 6.6 | 936 (29.0) | 926 (28.9) | 0.2 |  |
| Beta blockers | 2,884 (89.7) | 3,339 (89.3) | 1.3 | 2,887 (89.5) | 2,868 (89.5) | 0.1 |  |
| Calcium Channel blockers | 650 (20.2) | 838 (22.4) | 5.4 | 676 (21.0) | 675 (21.1) | 0.3 |  |
| Non-dihydropyridine CCB | 226 (7.0) | 288 (7.7) | 2.6 | 238 (7.4) | 236 (7.4) | 0.1 |  |
| Thiazide diuretics | 593 (18.5) | 767 (20.5) | 5.2 | 633 (19.6) | 636 (19.8) | 0.6 |  |
| Loop diuretics | 2,624 (81.6) | 3,163 (84.6) | 8.0 | 2,687 (83.3) | 2,663 (83.1) | 0.4 |  |
| Aldosterone antagonists | 1,173 (36.5) | 1,247 (33.4) | 6.6 | 1,128 (34.9) | 1,119 (34.9) | 0.1 |  |
| Digoxin | 609 (18.9) | 579 (15.5) | 9.2 | 559 (17.3) | 557 (17.4) | 0.2 |  |
| Hydralazine / Isosorbide | 734 (22.8) | 1,082 (29.0) | 14.0 | 829 (25.7) | 835 (26.1) | 0.9 |  |
| Other heart failure medications ^d^ | 419 (13.0) | 273 (7.3) | 19.0 | 320 (9.9) | 318 (9.9) | 0.0 |  |
| Statins | 2,731 (85.0) | 3,123 (83.6) | 3.9 | 2,711 (84.0) | 2,698 (84.2) | 0.5 |  |
| Anticoagulants | 1,435 (44.6) | 1,517 (40.6) | 8.2 | 1,361 (42.2) | 1,346 (42.0) | 0.3 |  |
| Antiplatelets | 1,125 (35.0) | 1,274 (34.1) | 1.9 | 1,113 (34.5) | 1,112 (34.7) | 0.4 |  |
| SGLT2: Sodium-glucose cotransporter-2 inhibitor; GLP-1RA: Glucagon-like peptide-1 receptor agonist; SD:  standardized difference; IPT: Inverse probability of treatment; ACE: Angiotensin converting enzyme; ARB:  Angiotensin II receptor blockers; CCB: calcium channel blocker  ^a^ Heart failure subtype was determined by requiring presence of ICD-10 codes: I50.2x or I50.3x for HFrEF and HFpEF respectively, in either the first or second position of a heart failure hospitalization. The positive predictive value for this approach exceeds 85% for identifying HFrEF and HFpEF patients.  ^b^ For ease of interpretation, the denominator of the DPP4i arm is weighted down to the SGLT2i group.  ^c^ Standardized differences greater than 10 imply a meaningful difference in the patient characteristic. After IPTW weighting, there were no differences that exceeded this threshold.  ^d^Include angiotensin receptor-neprilysin inhibitors and hyperpolarization-activated cyclic nucleotide-gated (HCN) channel blockers. | | | | | | |  |

| **Appendix Table 4: Baseline patient characteristics prior to after IPT weighting, SGLT2i v GLP-1RA among patients with heart failure with preserved ejection fraction ^a^** | | | | | | |  |
| --- | --- | --- | --- | --- | --- | --- | --- |
|  | **SGLT2i** | **GLP-1RA** | **SD ^c^** | **SGLT2i** | **GLP-1RA** | **SD ^c^** |  |
|  | **(n=3,578)** | **(n=5,475) ^b^** |  | **(n=3,578)** | **(n=3,578) ^b^** |  |  |
| **Sociodemographics** |  |  |  |  |  |  |  |
| Age, mean (Standard Deviation) | 73.0 (10.4) | 70.7 (10.6) | 15.5 | 71.3 (10.9) | 71.4 (10.5) | 0.6 |  |
| Male | 1,637 (45.8) | 2,125 (38.8) | 14.1 | 1,489 (41.4) | 1,487 (41.6) | 0.5 |  |
| Race, White | 2,726 (76.2) | 4,018 (73.4) | 6.5 | 2,692 (74.8) | 2,667 (74.7) | 0.4 |  |
| Race, Black | 373 (10.4) | 783 (14.3) | 11.8 | 461 (12.8) | 459 (12.8) | 0.1 |  |
| Other Race | 479 (13.4) | 674 (12.3) | 3.2 | 445 (12.4) | 446 (12.5) | 0.4 |  |
| Calendar year (2013, 2014,2015) | 634 (17.7) | 1,044 (19.1) | 3.5 | 696 (19.4) | 669 (18.7) | 1.6 | |
| Calendar year (2016, 2017) | 1,019 (28.5) | 1,717 (31.4) | 6.3 | 1,089 (30.3) | 1,082 (30.3) | 0.1 | |
| Calendar year (2018, 2019) | 1,925 (53.8) | 2,714 (49.6) | 8.5 | 1,813 (50.4) | 1,820 (51.0) | 1.2 | |
| **Diabetes related factors** |  |  | . |  |  | . |  |
| Metformin | 2,014 (56.3) | 2,325 (42.5) | 27.9 | 1,735 (48.2) | 1,714 (48.0) | 0.4 |  |
| Sulfonylureas | 1,462 (40.9) | 1,879 (34.3) | 13.5 | 1,356 (37.7) | 1,328 (37.2) | 1.0 |  |
| DPP4i | 1,159 (32.4) | 1,447 (26.4) | 13.1 | 1,033 (28.7) | 1,028 (28.8) | 0.2 |  |
| Insulin | 1,487 (41.6) | 3,566 (65.1) | 48.6 | 2,034 (56.5) | 2,003 (56.1) | 0.9 |  |
| Thiazolidinediones | 255 (7.1) | 277 (5.1) | 8.7 | 217 (6.0) | 212 (5.9) | 0.3 |  |
| Diabetes, ocular complications | 643 (18.0) | 1,250 (22.8) | 12.1 | 736 (20.5) | 743 (20.8) | 0.8 |  |
| Diabetes, renal complications | 1,300 (36.3) | 2,789 (50.9) | 29.8 | 1,605 (44.6) | 1,608 (45.0) | 0.8 |  |
| Diabetes, neurological complications | 1,639 (45.8) | 2,960 (54.1) | 16.6 | 1,851 (51.4) | 1,824 (51.1) | 0.7 |  |
| **Other factors** |  |  | . |  |  | . |  |
| Frailty | 599 (16.7) | 1,110 (20.3) | 9.1 | 683 (19.0) | 676 (18.9) | 0.1 |  |
| Myocardial infarction | 169 (4.7) | 367 (6.7) | 8.5 | 202 (5.6) | 210 (5.9) | 1.1 |  |
| Stroke | 47 (1.3) | 93 (1.7) | 3.2 | 53 (1.5) | 56 (1.6) | 0.8 |  |
| Peripheral vascular disease | 1,087 (30.4) | 1,714 (31.3) | 2.0 | 1,103 (30.6) | 1,103 (30.9) | 0.5 |  |
| Other ischemic heart disease | 2,570 (71.8) | 3,574 (65.3) | 14.1 | 2,410 (67.0) | 2,422 (67.8) | 1.8 |  |
| Renal insufficiency | 1,731 (48.4) | 3,492 (63.8) | 31.4 | 2,052 (57.0) | 2,052 (57.5) | 0.9 |  |
| ACE inhibitors | 1,405 (39.3) | 2,098 (38.3) | 1.9 | 1,424 (39.6) | 1,400 (39.2) | 0.8 |  |
| ARBs | 1,128 (31.5) | 1,638 (29.9) | 3.5 | 1,107 (30.8) | 1,088 (30.5) | 0.6 |  |
| Beta blockers | 2,814 (78.6) | 4,206 (76.8) | 4.4 | 2,759 (76.7) | 2,763 (77.3) | 1.6 |  |
| Calcium channel blockers | 1,196 (33.4) | 2,009 (36.7) | 6.9 | 1,269 (35.3) | 1,268 (35.5) | 0.5 |  |
| Non-dihydropyridine CCB | 528 (14.8) | 752 (13.7) | 2.9 | 510 (14.2) | 507 (14.2) | 0.0 |  |
| Thiazide diuretics | 847 (23.7) | 1,475 (26.9) | 7.5 | 930 (25.8) | 916 (25.6) | 0.5 |  |
| Loop diuretics | 2,913 (81.4) | 4,689 (85.6) | 11.4 | 3,017 (83.8) | 2,994 (83.8) | 0.0 |  |
| Aldosterone antagonists | 763 (21.3) | 1,197 (21.9) | 1.3 | 787 (21.9) | 773 (21.6) | 0.6 |  |
| Digoxin | 371 (10.4) | 429 (7.8) | 8.8 | 309 (8.6) | 313 (8.8) | 0.6 |  |
| Hydralazine / Isosorbide | 885 (24.7) | 1,493 (27.3) | 5.8 | 928 (25.8) | 939 (26.3) | 1.1 |  |
| Other heart failure medications ^d^ | 67 (1.9) | 65 (1.2) | 5.6 | 51 (1.4) | 51 (1.4) | 0.0 |  |
| Statins | 2,888 (80.7) | 4,359 (79.6) | 2.8 | 2,869 (79.7) | 2,855 (79.9) | 0.5 |  |
| Anticoagulants | 1,516 (42.4) | 2,064 (37.7) | 9.5 | 1,402 (38.9) | 1,408 (39.4) | 1.0 |  |
| Antiplatelets | 963 (26.9) | 1,391 (25.4) | 3.4 | 921 (25.6) | 924 (25.9) | 0.6 |  |
| SGLT2: Sodium-glucose cotransporter-2 inhibitor; GLP-1RA: Glucagon-like peptide-1 receptor agonist; SD:  standardized difference; IPT: Inverse probability of treatment; ACE: Angiotensin converting enzyme; ARB:  Angiotensin II receptor blockers; CCB: calcium channel blocker  ^a^ Heart failure subtype was determined by requiring presence of ICD-10 codes: I50.2x or I50.3x for HFrEF and HFpEF respectively, in either the first or second position of a heart failure hospitalization. The positive predictive value for this approach exceeds 85% for identifying HFrEF and HFpEF patients.  ^b^ For ease of interpretation, the denominator of the DPP4i arm is weighted down to the SGLT2i group.  ^c^ Standardized differences greater than 10 imply a meaningful difference in the patient characteristic. After IPTW weighting, there were no differences that exceeded this threshold.  ^d^Include angiotensin receptor-neprilysin inhibitors and hyperpolarization-activated cyclic nucleotide-gated (HCN) channel blockers. | | | | | | |  |

| **Appendix Table 5: Reasons for censoring in the study cohorts** | | |
| --- | --- | --- |
|  | ***SGLT2i vs DPP4i*** | |
|  | **HFrEF** | **HFpEF** |
| Exposure discontinuation | 7,931 (57.1) | 9,864 (56.4) |
| End of data | 5,536 (39.9) | 7,134 (40.8) |
| Exposure switching | 415 (3.0) | 495 (2.8) |
|  | ***SGLT2i vs GLP-1RA*** | |
|  | **HFrEF** | **HFpEF** |
| Exposure discontinuation | 3,920 (56.4) | 5,060 (55.9) |
| End of data | 2,684 (38.6) | 3,489 (38.5) |
| Exposure switching | 347 (5.0) | 504 (5.6) |
| SGLT2: Sodium-glucose cotransporter-2 inhibitor; DPP4i:  Dipeptidyl peptidase 4 inhibitor; GLP-1RA: Glucagon-like peptide-1 receptor agonist | | |

| **Appendix Table 6: Risk of cardiovascular outcomes among patients initiating SGLT2i compared to other therapies, by heart failure subtype: Sensitivity and secondary analysis.** | | | | | | |
| --- | --- | --- | --- | --- | --- | --- |
|  | **Unadjusted** | | **IPTW adjusted HR (95% CI)** | **Unadjusted** | | **IPTW adjusted HR (95% CI) ^b^** |
|  | **No. events (IR) ^a^** | |  | **No. events (IR) ^a^** | |  |
|  | ***Heart Failure with Reduced Ejection Fraction*** | | | ***Heart Failure with Preserved Ejection Fraction*** | | |
|  | **SGLT2i** | **DPP4i** |  | **SGLT2i** | **DPP4i** |  |
|  | **(n=2,503)** | **(n=11,379)** |  | **(n=2,846)** | **(n=14,647)** |  |
| **Other secondary outcomes** |  |  |  |  |  |  |
| MI hospitalizations | 116 (7.0) | 1,065 (10.8) | 0.81 (0.69, 0.95) | 139 (7.3) | 1,343 (10.4) | 0.88 (0.76, 1.02) |
| Stroke hospitalizations | 30 (1.8) | 216 (2.1) | 1.02 (0.74, 1.41) | 33 (1.7) | 333 (2.5) | 0.90 (0.68, 1.21) |
| All-cause mortality | 426 (11.6) | 4,987 (22.3) | 0.39 (0.34, 0.46) | 468 (10.7) | 6,227 (22.2) | 0.46 (0.40, 0.52) |
| **MI, stroke or HF hospitalizations** | 719 (53.7) | 5,645 (82.8) | 0.88 (0.82, 0.95) | 831 (53.5) | 7,262 (78.2) | 0.91 (0.85, 0.97) |
| **Intention to treat analyses** |  |  |  |  |  |  |
| HF hospitalizations | 1,119 (48.2) | 7,716 (65.9) | 0.79 (0.74, 0.83) | 1,297 (47.3) | 9,784 (67.9) | 0.76 (0.72, 0.80) |
| MI or stroke hospitalizations | 344 (13.2) | 2,069 (14.0) | 1.09 (1.00, 1.20) | 391 (12.7) | 2,966 (14.5) | 1.00 (0.92, 1.11) |
|  | **SGLT2i** | **GLP-1RA** |  | **SGLT2i** | **GLP-1RA** |  |
|  | **(n=3,214)** | **(n=3,737)** |  | **(n=3,578)** | **(n=5,475)** |  |
| **Other secondary outcomes** |  |  |  |  |  |  |
| MI hospitalizations | 146 (6.7) | 242 (8.7) | 1.01 (0.83, 1.23) | 169 (6.9) | 367 (8.4) | 0.96 (0.80, 1.14) |
| Stroke hospitalizations | 36 (1.6) | 49 (1.7) | 0.97 (0.63, 1.50) | 47 (1.9) | 93 (2.1) | 0.94 (0.67, 1.33) |
| All-cause mortality | 584 (11.9) | 877 (13.2) | 0.86 (0.72, 1.03) | 676 (11.9) | 1,185 (12.1) | 0.94 (0.80, 1.10) |
| **MI, stroke or HF hospitalizations** | 908 (50.4) | 1,336 (61.9) | 0.98 (0.90, 1.06) | 1,092 (55.4) | 2,056 (62.4) | 0.96 (0.89, 1.03) |
| **Intention to treat analyses** |  |  |  |  |  |  |
| HF hospitalizations | 1,444 (44.7) | 2,083 (54.3) | 0.89 (0.84, 0.95) | 1,697 (48.3) | 3,079 (54.5) | 0.91 (0.86, 0.97) |
| MI or stroke hospitalizations | 442 (12.7) | 660 (14.5) | 0.99 (0.88, 1.12) | 512 (12.8) | 2,056 (62.4) | 1.00 (0.90, 1.11) |
| SGLT2i: Sodium-glucose cotransporter-2 inhibitor; DPP4i: dipeptidyl peptidase 4 inhibitor; GLP-1RA: Glucagon-like peptide-1 receptor agonist; CI: confidence intervals; IR: incidence rate; HR: hazard ratio  ^a^ Represent the unadjusted number of events and incidence rates per 100 person-years  ^b^Hazard ratios adjusted for variables described in Table 1 using stabilized inverse probability of treatment weighting | | | | | | |
